# MicroRNA Dysregulation, Gene Networks, and Risk for Schizophrenia in 22q11.2 Deletion Syndrome

**DOI:** 10.3389/fneur.2014.00238

**Published:** 2014-11-21

**Authors:** Daniele Merico, Gregory Costain, Nancy J. Butcher, William Warnica, Lucas Ogura, Simon E. Alfred, Linda M. Brzustowicz, Anne S. Bassett

**Affiliations:** ^1^The Centre for Applied Genomics and Program in Genetics and Genome Biology, The Hospital for Sick Children, Toronto, ON, Canada; ^2^Clinical Genetics Research Program, Centre for Addiction and Mental Health, Toronto, ON, Canada; ^3^Institute of Medical Science, University of Toronto, Toronto, ON, Canada; ^4^Department of Genetics and the Human Genetics Institute of New Jersey, Rutgers University, Piscataway, NJ, USA; ^5^The Dalglish Family Hearts and Minds Clinic for 22q11.2 Deletion Syndrome, Toronto General Hospital, University Health Network, Toronto, ON, Canada; ^6^Department of Psychiatry, Toronto General Research Institute, University Health Network, Toronto, ON, Canada; ^7^Department of Psychiatry, University of Toronto, Toronto, ON, Canada

**Keywords:** DiGeorge syndrome, 22q11.2, 22q11 deletion, schizophrenia, DGCR8, miRNA, protein interaction network, synapse

## Abstract

The role of microRNAs (miRNAs) in the etiology of schizophrenia is increasingly recognized. Microdeletions at chromosome 22q11.2 are recurrent structural variants that impart a high risk for schizophrenia and are found in up to 1% of all patients with schizophrenia. The 22q11.2 deletion region overlaps gene *DGCR8*, encoding a subunit of the miRNA microprocessor complex. We identified miRNAs overlapped by the 22q11.2 microdeletion and for the first time investigated their predicted target genes, and those implicated by *DGCR8*, to identify targets that may be involved in the risk for schizophrenia. The 22q11.2 region encompasses seven validated or putative miRNA genes. Employing two standard prediction tools, we generated sets of predicted target genes. Functional enrichment profiles of the 22q11.2 region miRNA target genes suggested a role in neuronal processes and broader developmental pathways. We then constructed a protein interaction network of schizophrenia candidate genes and interaction partners relevant to brain function, independent of the 22q11.2 region miRNA mechanisms. We found that the predicted gene targets of the 22q11.2 deletion miRNAs, and targets of the genome-wide miRNAs predicted to be dysregulated by DGCR8 hemizygosity, were significantly represented in this schizophrenia network. The findings provide new insights into the pathway from 22q11.2 deletion to expression of schizophrenia, and suggest that hemizygosity of the 22q11.2 region may have downstream effects implicating genes elsewhere in the genome that are relevant to the general schizophrenia population. These data also provide further support for the notion that robust genetic findings in schizophrenia may converge on a reasonable number of final pathways.

## Introduction

MicroRNAs (miRNAs) are small non-coding RNAs that regulate gene expression at the level of translation of messenger RNA to protein (1, 2). A recent review documents the ever increasing number of miRNAs identified throughout the human genome and the emerging knowledge about their target genes (2). Individual miRNAs can target multiple messenger RNAs, effectively controlling expression of a suite of genes. Thus, the alteration of a single miRNA with respect to its genomic sequence, copy number, and/or expression can have broad implications for both normal development and cellular function throughout life. Also, a single gene’s messenger RNA can be targeted for modulation by several miRNAs. Mature miRNAs are processed from double-stranded primary miRNA transcripts by a microprocessor complex comprised of two main cofactors: the RNA-binding protein Pasha, encoded by the *DGCR8* gene located within the typical 22q11.2 deletion region, and Drosha, the endonuclease responsible for cleaving RNA (1). The prominent role of *DGCR8* in miRNA-processing implicates a miRNA mechanism in individuals with 22q11.2 deletion syndrome (22q11.2DS; OMIM #188400/#192430). 22q11.2DS is the most common genomic disorder in humans (3–5). The highly variable expression of the underlying 22q11.2 microdeletion has led in the past to various names being applied to the same condition, including DiGeorge and velocardiofacial syndromes (3). The penetrance for at least one major feature is high, including cardiac and palatal anomalies, endocrine disorders, intellectual and learning disabilities, and schizophrenia and other neuropsychiatric diseases (3). Notably, the 22q11.2 microdeletion is the single greatest known molecular risk factor for schizophrenia (6, 7). Up to 1% of patients with schizophrenia have a 22q11.2 deletion associated with 22q11.2DS (8), and individuals with 22q11.2DS have a 20–25% lifetime risk of schizophrenia (i.e., a ~25-fold elevation over the general population risk) (7).

Multiple lines of evidence implicate miRNAs in the etiology of schizophrenia in the general population. These include altered miRNA expression profiles compared with controls in postmortem studies of brain tissue (9–16) and peripheral serum samples (17, 18). Also, recent data suggest an enrichment of rare copy number variations (CNVs) overlapping miRNAs in schizophrenia compared to a control population, even after controlling for CNV size and excluding 22q11.2 deletions (19).

In individuals with typical 22q11.2 deletions, there is preliminary evidence of a unique miRNA expression profile (20), and there are data from mouse models implicating an effect of reduced *Dgcr8* copy number on global miRNA expression (21–26). We have previously proposed a theory that mechanistically explains the link between 22q11.2DS, miRNAs, and schizophrenia risk, with evidence from an adult 22q11.2DS sample that the 22q11.2 deletion may unmask effects of variants in genes across the genome (27). In the current study, we hypothesized that the effect of a 22q11.2 deletion on phenotypic expression of schizophrenia could involve not only the hemizygosity of *DGCR8* but also reduced dosage of all of the miRNAs within the 22q11.2 deletion region. We identified all putative miRNAs in the 22q11.2 deletion region and investigated functional enrichment profiles of their predicted target genes. We then explored the role of these targets, also considering those of miRNAs outside of the 22q11.2 region reported to be dysregulated by hemizygosity of *DGCR8*, in a newly created protein interaction network composed of schizophrenia candidate genes and interaction partners relevant to brain function.

## Materials and Methods

### Genome build

We relied on the latest genome build (GRCh38/hg20), used in the June/July 2014 release of the miRBase resource (28, 29), to generate a comprehensive list of 22q11.2 deletion region miRNAs and to analyze miRNA density in the rest of the genome. Because many gene information resources have not yet been updated to the latest assembly, we used genome build GRCh37/hg19 for all gene-related methods, including miRNA target gene prediction and derivation of the schizophrenia gene interaction network.

### 22q11.2 deletion region miRNA annotation and genome-wide density calculations

**Figure 1:**
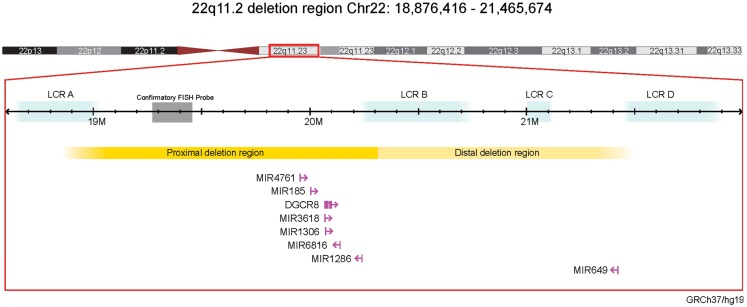
**Schematic of miRNAs and the miRNA-processing gene *DGCR8* in the 22q11.2 deletion region (genome build GRCh37/hg19)**. Affymetrix Human SNP Array 6.0 22q11.2 deletion breakpoints are shown (31). Gene and miRNA sizes are enlarged for illustrative purposes and are not to scale. FISH, fluorescence *in situ* hybridization; LCR, low copy repeats.

We annotated the typical 2.6 Mb 22q11.2 deletion region (chr22:18,876,416–21,465,674 [hg19]; Affymetrix Human SNP Array 6.0 breakpoint) for miRNA-related genomic content, using miRBase 21 (accessed in August 2014) to identify all putative miRNAs in the region (28, 29) and RefSeq (30) to map *DGCR8* and these miRNAs to the region of interest (Figure 1). To assess miRNA density within this 22q11.2 deletion region and compare to miRNA density in the rest of the genome, for each miRNA in miRBase 21, we counted how many miRNA primary transcripts were contained in a symmetric 2.6 Mb region around the miRNA transcript start (i.e., within 1.3 Mb on either side). Note that there is a one-to-one correspondence between miRBase 21 miRNA genes and miRNA primary transcripts, even in the presence of a duplicate miRNA gene at different genomic loci; primary transcripts are precursors of mature miRNAs.

### Prediction of miRNA target genes

We employed a conservative strategy to examine the predicted target genes of 22q11.2 deletion region miRNAs (22). We first selected two well-established target prediction tools: TargetScan 6.2 (32) and DIANA microT-CDS (33). For each miRNA of interest, we separately retrieved targets for the -3p and -5p miRNA mature forms, if available (Table 1). For each prediction method, gene prediction scores were transformed into percentiles considering predictions for all putative targets and all miRNAs (e.g., if a gene has score *x* for miRNA *m*, and *x* corresponds to the top 10% of all scores for that prediction method, the corresponding percentile-transformed score *x*’ is 0.10). Then, for each of the six 22q11.2 miRNAs with available targets from both tools (i.e., all but miR-6816; Table 1), we generated a final ranking based on the average percentile-transformed score from the two prediction tools. Whenever a gene was a target of both the -5p and -3p forms, its percentile-transformed scores for the two forms were also averaged (applicable to miR-4761 TargetScan and microT predictions, and miR-185 TargetScan predictions). Based on this final ranking, for each miRNA we compiled three lists of the highest-ranked predicted gene targets: the top 200, the top 400, and the top 800. For miR-1306, only 149 targets were retrieved and used in all subsequent analyses.

**Table 1:** **Characteristics of microRNAs (miRNAs) encoded within the 22q11.2 microdeletion region (current to August 2014)**.

miRNA primary coding sequence		Supporting miRNA evidence and quality							Target genes^e^	
miRNA	Coordinates [hg19]	Pub Med No.	miRBase NGS		Other evidence (miRBase)	GeneCards quality score^b^	Brain expression^c^	Conservation^d^	Predicted target genes	Mean target gene score
			Exp. No.	Read No.					
miR-185^a^	chr22:20020662-20020743	27	76	48635	Cloned	3	Yes	Primate, murine	Yes	0.042062
miR-649	chr22:21388465-21388561	1	3	3	RT-PCR, SAGE	8	No	Primate	Yes	0.099593
miR-1286	chr22:20236657-20236734	1	19	328	None	8	Yes	Primate	Yes	0.058791
miR-1306^a^	chr22:20073581-20073665	1	61	1329	None	3	Yes	Primate, murine	Yes	0.404076
miR-3618	chr22:20073269-20073356	1	8	9	None	3	No	Murine	Yes	0.068121
miR-4761^a^	chr22:19951276-19951357	1	12	26	None	3	–	None	Yes	0.080270
miR-6816^a^	chr22:20102209-20102274	1	4	5	None	2	–	None	No	N/A

*miRNA, miRNAs encoded within the 22q11.2 region (chr22:18876416-21465674 [hg19]); coordinates [hg19], miRNA primary transcript coordinates retrieved from miRBase (release 21) (29); PubMed No., number of PubMed articles retrieved from miRBase (29); Exp No., number of next-generation sequencing (NGS) experiments retrieved from miRBase (29); Read No., read counts from NGS experiments retrieved from miRBase (29); RT-PCR, reverse transcription-polymerase chain reaction; SAGE, serial analysis of gene expression*.

*^a^3p and 5p mature miRNA species identified*.

*^b^Indicates how many RNA databases have information about this miRNA, and whether the miRNA is expressed or is known to be functional (score ≥5 indicates miRNA is known to be expressed, score ≥10 indicates gene is known to be functional; www.genecards.org)*.

*^c^Human prefrontal cortex and/or cerebellar expression (34); mir-4761 and mir-6816 were unavailable for this publication*.

*^d^Limited to non-human primate and murine homologs; data collated from miRBase, Ensembl, and NCBI Entrez Gene*.

*^e^Availability of predicted target genes for mature miRNAs with data available using two standard prediction tools (TargetScan and microT-CDS), and average percentile-transformed scores for the 200 top-ranked predicted target genes per miRNA; 149 genes for mir-1306. See text for further details*.

### miRNA functional enrichment analysis

Functional enrichment of the miRNAs from the 22q11.2 deletion region was ascertained by testing if a given functional gene-set (see below) had more genes targeted by the 22q11.2 deletion region miRNAs than collectively for all other miRNAs in the genome, using the same target prediction methods as for the 22q11.2 deletion miRNAs. The background set of all miRNA targets consisted of 14,192 genes. This approach is robust to functional biases, such as miRNA targets being collectively enriched in specific functions. Of note, we tested the background set of all miRNA targets and indeed found enrichment in functions such as development, cell cycle, and transcriptional regulation. We tested miRNA target functional enrichment using the one-tailed Fisher’s Exact Test. Specifically, we constructed a contingency table for each miRNA and gene-set pair (*miRNA*_*i*, *GS*_*j*), with the following gene counts: (i) target genes of *miRNA*_*i* also found in functional gene-set *GS*_*j*, (ii) target genes of *miRNA*_*i* found in other functional gene-sets but not in *GS*_*j*, (iii) target genes of other miRNAs but not of *miRNA*_*i* found in functional gene-set *GS*_*j*, and (iv) target genes of other miRNAs but not of *miRNA*_*i* found in other functional gene-sets but not in *GS*_*j*. Official gene symbols from miRNA target prediction tools were converted to Entrez Gene ID for the enrichment analysis using the Bioconductor package org.Hs.eg.db version 2.14.0^1^.

The functional gene-sets used for enrichment analyses were derived from: (a) Gene Ontology annotations (GO) (35), (b) Kyoto Encyclopedia of Genes and Genomes (KEGG) (36), Reactome (37), National Cancer Institute (NCI) (38), and BioCarta^2^ pathway databases (downloaded 17 October 2013). Only gene-sets with sizes between 15 and 900 genes were used; in our experience, larger gene-sets are less informative and smaller gene-sets are detrimental for multiple test correction. This resulted in 5,794 gene-sets. To control for multiple testing, we used the Benjamini–Hochberg False Discovery Rate (FDR) method. All statistical analyses were performed using R 3.1.1 software.

### Visualization of miRNA functional enrichment results

For each of the six 22q11.2 deletion region miRNAs with target genes, we retained only the gene-sets that were significant at a nominal *p*-value threshold of 0.01 in at least two of the three target gene thresholds used (top 200, top 400, and top 800). For miR-1306, where only 149 targets could be retrieved, we used a more stringent significance threshold (nominal *p*-value ≤ 0.005). Functional gene-sets passing these significance filters were visualized using the Cytoscape plugin Enrichment Map (version 1.2) (39), setting the Jaccard and overlap combined coefficient threshold to 0.225. Circles were colored based on the miRNA that had gene targets enriched in the corresponding gene-set. Functional clusters were manually identified and labeled.

### Placing 22q11.2 deletion region miRNA-related gene targets in the context of a schizophrenia gene network

We then attempted to identify which genes predicted to be implicated by a 22q11.2 deletion region miRNA-related mechanism may contribute to schizophrenia etiology. To this end, we considered both the potential effects on gene targets related to the reduced dosage of 22q11.2 region miRNAs, and the potential effects related to reduced dosage of *DGCR8* and the associated changes in miRNA-processing genome-wide. Although many studies have investigated the rare and common variants contributing to schizophrenia etiology, it is reasonable to assume that many genes and genetic mechanisms remain undiscovered (40). To address this issue, we first constructed a protein interaction network of schizophrenia candidate genes and interaction partners relevant to brain function (details below). We then mapped the target genes derived from 22q11.2 deletion region miRNA-related mechanisms onto this schizophrenia network.

### Development of a gene list relevant to schizophrenia

Schizophrenia candidate genes were curated from sources that employed two different approaches. First, from the recent genome-wide case-control association study (GWAS) of schizophrenia by the Psychiatric Genomics Consortium (PGC), a type of study able to detect common sequence-based variants with low effect sizes in very large samples, we selected the 35 genes the authors highlighted in their supplementary information as those of particular interest for schizophrenia (40). Second, we considered the 107 genes of interest highlighted in our previous case-control study of rare CNV in a community schizophrenia sample, in which 22q11.2 deletions were *a priori* excluded (41). Each gene was overlapped by one or more very rare CNVs (i.e., gene dosage effects with potential high effect sizes) and was of potential relevance to brain function based on results of systematic searches of human (e.g., Online Mendelian Inheritance in Man)^3^ and model organism (e.g., Mouse Genome Informatics)^4^ databases (41). As annotated elsewhere (41), many of these 107 genes had also been implicated in other studies of rare copy number or sequence variation (42–54). Combining these two lists resulted in 141 genes, of which 139 were successfully mapped to Entrez Gene identifiers. One gene was present in both lists (*GRIN2A*).

We then defined three criteria to establish if a gene is relevant to brain function: (i) human brain expression level, (ii) gene function, and (iii) mouse neurological or neurodevelopmental phenotype. For human brain expression, we selected 9,199 genes displaying higher than average gene expression in any brain region and developmental time point present in the BrainSpan data-set (downloaded September 2012) (55). For gene function, we selected 3,192 genes found in any of the following gene-sets: (i) human GO central nervous system development, (ii) human GO neuron development, (iii) human GO synapse, (iv) human GO neuron projection, (v) human GO neuron cell body, (vi) human post-synaptic density components detected by proteomics (56), and (vii) human orthologs of mouse FMR1 targets detected experimentally in neurons (57). For mouse phenotypes, we selected 3,479 human orthologs of mouse genes with a reported neurobehavioral or abnormal nervous system phenotype (Mouse Genome Informatics; downloaded August 2013)^4^. It was previously shown that these gene-sets display significantly higher rare variant burden in neuropsychiatric disorders (41, 44, 58–61). By requiring at least one of the three criteria to be met, we retained 118 candidate genes. All the 21 initial candidate genes that were excluded came from the CNV study. In addition, by requiring at least two of the three criteria to be met, we labeled 3,813 other genes as relevant to brain function.

### Schizophrenia network construction and visualization

To construct a schizophrenia gene network, we first imported protein–protein interactions from the GeneMANIA website (August 2014) (62), derived from BioGRID (63), Intact (64), Bind (65), Dip (66), and HPRD databases (67). We retained only those interactions with GeneMANIA weight ≥0.02 to minimize false positives; this threshold was chosen based on manual inspection of the data and overall interaction count. This resulted in 90,188 unique interactions among 14,091 genes.

Genes relevant to brain function were additively scored based on their first-degree interaction as follows: (i) −1 for every interaction with a gene not labeled as relevant to brain function or as a schizophrenia candidate; (ii) +0.5 for every interaction with a gene labeled as relevant to brain function (excluding schizophrenia candidates; 3,813 genes); and, (iii) +2.5 for every interaction with a schizophrenia candidate gene (118 genes). We evaluated slightly different scoring schemes and found very similar gene rankings (data not shown). We retained genes relevant to brain function with interaction score ≥1.5. We constructed the final network by including all interactions with interaction partners corresponding to genes relevant to brain function and schizophrenia candidates but not other genes, following the principle of “guilt-by-association” (68). The network was visualized in Cytoscape 2.8.2 using the spring-embedded layout.

### Mapping of genes influenced by 22q11.2 deletion region miRNA-related mechanisms on a schizophrenia network

In order to map the target genes influenced by 22q11.2 deletion region miRNA-related mechanisms onto the schizophrenia network, we first studied the total 1,081 predicted target genes of the six 22q.11.2 miRNAs. For the *DGCR8*-related mechanism, we used the 3,022 genes that were amongst the top 200 targets of at least two miRNAs differentially expressed in the mouse model of *DGCR8* haploinsufficiency (21), and not targeted by any of the six 22q11.2 miRNAs. We compared each of these target sets with the 449 genes in the schizophrenia network, using the one-sided Fisher’s Exact Test to assess the significance of the overlap.

## Results

### miRNAs in the 22q11.2 deletion region

There were seven putative miRNAs encoded within the typical 22q11.2 deletion region (Table 1; Figure 1), corresponding to 11 mature (processed) miRNAs. All but one (miR-649) are in the nested proximal deletion region (Figure 1). The 22q11.2 deletion region is characterized by high miRNA density: the number of miRNA primary transcripts within this 2.6 Mb window is greater than the number found in 73.3% of same-sized regions surrounding other miRNA loci genome-wide. If one considered the six miRNAs in the 1.4 Mb proximal 22q11.2 deletion region, this miRNA density would be even more striking. Notably, miR-1306 and miR-3618 are just 309 bp apart and are encoded in the genomic sequence of *DGCR8*.

Considering the totality of experimental evidence supporting the existence of these putative miRNAs in the chromosome 22q11.2 region, miR-185 and miR-649 are the most established (Table 1). miR-1286 and miR-1306 have very good evidence based on next-generation sequencing (NGS) experiments but lack independent validation, while miR-3618 and miR-4761 have weaker NGS-based evidence (Table 1). The existence of miR-6816 is supported by limited evidence and there are no predicted genes targets at present (Table 1). Therefore, this miRNA was excluded from the analyses that follow. We note that, in general, supporting evidence is strongest for the lower numbered miRNAs, as these were discovered first, and far less for higher numbered miRNAs.

### Predicted gene targets of 22q11.2 region miRNAs are enriched for neurodevelopmental functions

Overall, the average percentile-transformation prediction scores for the top 200 target genes per miRNA were similar for each of the miRNAs in the 22q11.2 deletion region, with the exception of miR-1306 where there were just 149 target genes (Table 1). Because the percentile transformation is based on prediction scores for all miRNAs (see Materials and Methods), this indicates similarly well predicted (i.e., top 10%) targets for five of the six miRNAs in this region for their top 200 target genes.

**Figure 2:**
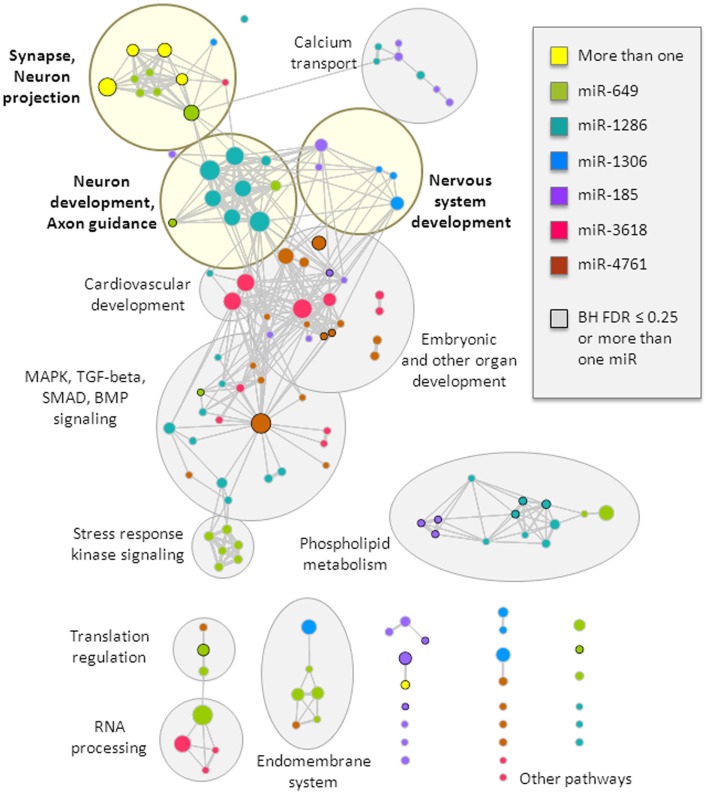
**Top functional gene-sets enriched in predicted targets of miRNAs from the 22q11.2 deletion region**. Enriched gene-sets are visualized as a network, where circles represent gene-sets and connections (lines) depict gene-set overlap; in this way, gene-sets that have a similar gene composition cluster into functional groups, which were manually identified and labeled (light yellow shades if neurobiologically related, and gray shades otherwise; more details can be found in Table S1 in Supplementary Material). Circle colors represent gene-set enrichment of results for six different miRNAs, with yellow representing enrichment in more than one miRNA; black circle border denotes enrichment Benjamini–Hochberg FDR ≤ 25% or enrichment in more than one miRNA. Circle size relates to the total number of genes in the gene-set. This visualization was created using the Cytoscape plugin Enrichment Map (version 1.2).

The target genes of these miRNAs were tested for enrichment in functional gene-sets. We retained only the gene-sets that were significant at a nominal *p*-value threshold of 0.01 in at least two of the three target gene thresholds used (top 200, top 400, and top 800). We identified several functional clusters of interest (Figure 2, more details are provided in Table S1 in Supplementary Material). In particular, the synapse and neuron projection cluster displayed enrichment that involved targets of more than one miRNA from the 22q11.2 deletion region (miR-649 and either miR-1286 or miR-185). All miRNAs, except miR-4761, displayed enrichment in at least one gene-set related to brain function or development, with specific clusters related to: (i) synapse and neuron projection components; (ii) nervous system development; and (iii) neuron development and axon guidance (Figure 2). In addition, targets of four miRNAs (miR-185, miR-1286, miR-3618, and miR-4761) displayed enrichment in other developmental processes and pathways, including embryonic development, the mitogen-activated protein kinase (MAPK) cascade, the bone morphogenetic protein (BMP) group of growth factors, and SMAD and transforming growth factor-beta (TGF-beta) signaling. Notably, miR-3618 targets displayed particular enrichment in cardiovascular development. These latter findings may be of interest with regards to other features of the typical 22q11.2DS phenotype, such as congenital cardiac defects (3).

### Schizophrenia candidate gene network and overlap of 22q11.2 deletion region miRNA targets

**Figure 3:**
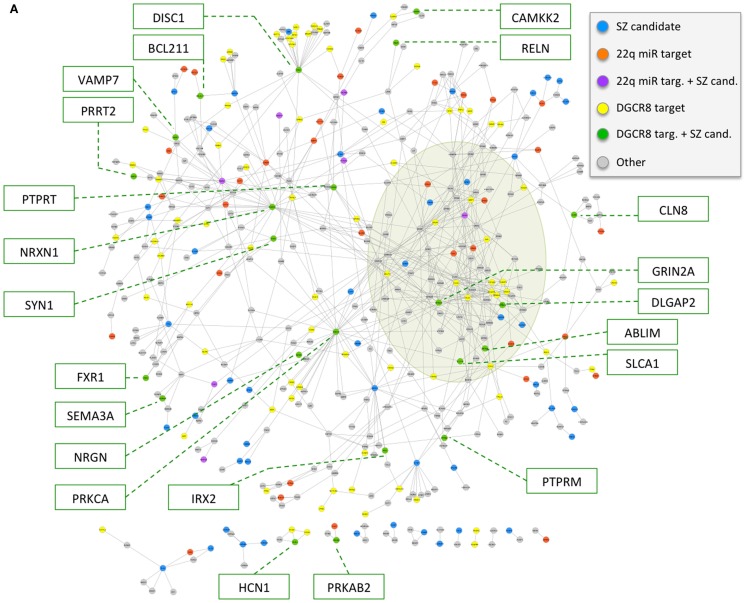

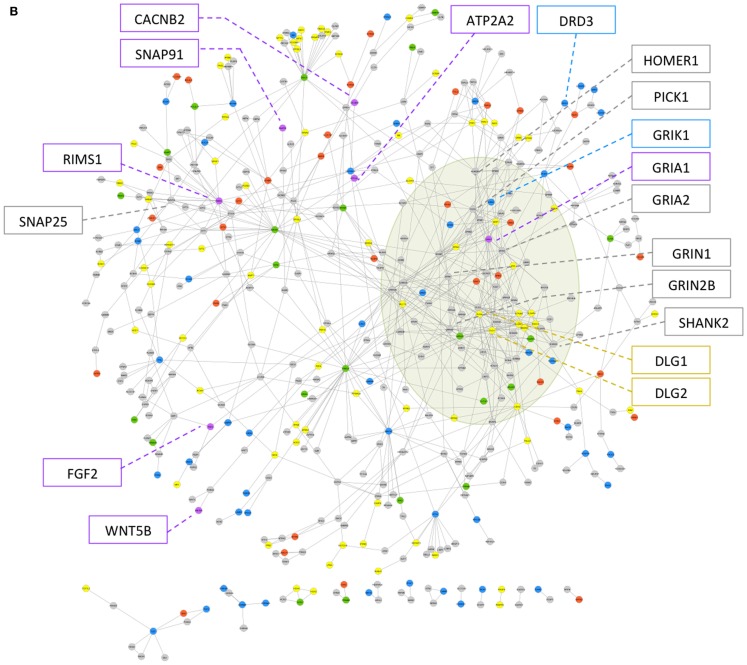
**(A,B)** Overlap of 22q11.2 deletion region miRNA-related mechanisms with a physical protein–protein interaction network of schizophrenia candidate genes and interaction partners relevant to brain function. The network is composed of 449 genes (78 schizophrenia candidates and 371 interaction partners relevant to brain function; see text and Table S2 in Supplementary Material), depicted as circles, and 773 physical protein–protein interactions, depicted as connecting lines. Genes are colored according to the overlap with 22q11.2 deletion region miRNA deregulation mechanism: (i) predicted targets of the six 22q11.2 miRNAs have color violet if they overlap with curated schizophrenia candidates used to seed the network, and orange otherwise; (ii) predicted targets of at least two miRNAs down-regulated in a model of decreased *DGCR8* processing mechanism, excluding predicted targets of the six 22q11.2 miRNAs, have color green if they overlap with curated schizophrenia candidates used to seed the network, and yellow otherwise. Other schizophrenia candidate genes have color blue, while the remainder of the genes have color gray. The densely connected network core corresponding to GRIAs, GRIKs, GRINs, DLGs, DLGAPs, SHANKs, and HOMERs is highlighted by an olive green shade. Gene names are highlighted in boxes for 22q11.2 deletion region miRNA target genes that overlap schizophrenia candidate genes **(A)**, and for target genes of miRNAs implicated by a decreased *DGCR8* processing mechanism that overlap schizophrenia candidate genes, as well as a selected subset of other genes of interest for schizophrenia **(B)**.

To investigate the potential role of 22q11.2 deletion region miRNA mechanisms in the high risk for schizophrenia associated with 22q11.2DS, we derived a schizophrenia gene network using methods unrelated to either miRNA mechanisms or the 22q11.2 deletion. The network was constructed on the basis of physical protein–protein interactions between schizophrenia candidates and genes deemed relevant to brain function; in particular, genes from the latter group were also required to have specific connectivity to schizophrenia candidates or other genes relevant for brain function (see Materials and Methods for details). The final network comprised 449 genes (78 of the 118 initial schizophrenia candidates and 371 of the initial 3,813 genes relevant to brain function) (Table S2 in Supplementary Material), with 773 interactions (Figure 3). The network is characterized by a densely connected core corresponding to glutamatergic ionotropic receptors (*GRIA*s, *GRIK*s, and *GRIN*s) and post-synaptic density organizers (*DLG*s, *DLGAP*s, *SHANK*s, and *HOMER*s). The network also includes key neuronal adhesion molecules (such as neurexins and neuroligins), components of the dopaminergic synapse (such as *DRD2* and *DRD3*), axon guidance molecules and receptors (such as ephrins, plexins, and netrins), and signaling hubs (such as *PRKCA*). While the candidate genes used to construct this network were selected using evidence for disease implication derived from two different approaches (40, 41), the network includes many additional genes of potential relevance to schizophrenia (e.g., *GRIA2*, *GRIN1*, *GRIN2B*, *HOMER1*, *PICK1*, and *SNAP25*).

We then used this network to investigate the two 22q11.2 deletion region miRNA-related mechanisms under study. We successfully mapped onto the schizophrenia network 40 of the total 1,081 predicted target genes of the six 22q11.2 miRNAs. We also mapped 100 of 3,022 genes implicated in a mouse model of *DGCR8* haploinsufficiency (21) and that were not targeted by the six 22q11.2 miRNAs. Both of these target sets exhibited a statistically significant overlap with the schizophrenia gene network (one-sided Fisher’s Exact Test *p*-value <0.05, odds-ratio point estimate = 1.4 and 1.3, respectively), and an apparently even distribution across the network (Figure 3).

Notably, upon ranking network genes by our network interaction score (see Materials and Methods) we found that 24 (48%) of the top-scoring 50 genes (Table S2 in Supplementary Material, Network Attributes, Network score) were implicated as targets of 22q11.2 region miRNAs or miRNAs dysregulated by *DGCR8* haploinsufficiency. These included schizophrenia candidate genes (*GRIA1*, *RIMS1*, *DLGAP2*, *GRIN2A*, and *NRXN1*) and several interaction partners. Amongst the latter were genes involved in glutamate and/or dopamine synapses (e.g., *GRIK2* and *GRM5*), and other genes relevant to brain function such as *NLGN2* and *STXBP1* (Table S2 in Supplementary Material). With respect to the total 78 schizophrenia candidate genes used to construct the network, other genes that were predicted targets of the 22q.11.2 region miRNAs were: *ATP2A2*, *CACNB2*, *FGF2*, *SNAP91*, and *WNT5B*. Included amongst the 22 candidate genes implicated by the *DGCR8* mechanism were *DISC1*, *RELN*, and *SYN1*.

## Discussion

In this study, we identified all putative miRNAs in the 22q11.2 deletion region and systematically investigated their predicted target genes. The functional enrichment profiles of their predicted targets suggested a role in neuronal processes and broader developmental pathways. We found that the genes targeted by these miRNAs, as well as the genes targeted by miRNAs outside of the 22q11.2 region yet predicted to be dysregulated because of the *DGCR8* hemizygous deletion (21), were significantly represented in a protein interaction network composed of schizophrenia candidate genes (40, 41) and brain-specific interaction partners. These results further our understanding of the potential pathway(s) from genotype to phenotype for the greatest known molecular risk factor for schizophrenia – a 22q11.2 microdeletion. The findings also provide insight into the etiology of schizophrenia more generally, and highlight the usefulness of studying the more genetically homogeneous model for schizophrenia provided by 22q11.2DS-schizophrenia.

### miRNA-mediated disease expression in 22q11.2DS and related genomic disorders

From the discovery of the first miRNA in *C. elegans* in 1993 (69) to the 2014 release of the online repository miRBase 21^5^, 1,881 precursor and 2,588 mature human miRNAs have been identified (29). This represents over a fourfold increase in numbers just since 2007 (2), and more miRNAs are likely to be discovered (70). These ongoing advances in our understanding of human genome regulation necessitate a critical reappraisal of the traditional protein-coding gene focus of cytogenetics. It is now appreciated that miRNA mechanisms may contribute to the pathogenesis of diverse neurodevelopmental and neurodegenerative disorders (2). Our results suggest that the 22q11.2 deletion region is characterized by increased miRNA density, and that target genes of these miRNAs are enriched in gene-sets of brain function and/or development, as well as other developmental processes and pathways. In 22q11.2DS, this provides an enhanced model for understanding multisystem pathogenesis that moves beyond *DGCR8*, *TBX1*, and other protein-coding genes within the 22q11.2 deletion region. The pleiotropy of the implicated miRNA target genes could contribute to the variable expression that is a hallmark of 22q11.2DS, and involve the brain and multiple other organs. This could help to explain the constellation of congenital features previously known as DiGeorge syndrome in addition to individual major later-onset features such as schizophrenia. Some of the classic variability associated with other “contiguous gene syndromes” could similarly be mediated by miRNA dysregulation and disruption of non-coding elements.

Consistent with our hypothesis, we found evidence that the effect of a 22q11.2 deletion on phenotypic expression of schizophrenia could involve not only the hemizygosity of *DGCR8* but also reduced dosage of all of the miRNAs within the 22q11.2 deletion region. Target genes of the 22q11.2 region miRNAs exhibited a statistically significant overlap with a protein interaction network composed of schizophrenia candidate genes and interaction partners relevant to brain function. These data are consistent with, and expand on, previous studies implicating miR-185 and its downstream pathways in schizophrenia (71). We also observed a statistically significant overlap between target genes of miRNAs predicted to be dysregulated by hemizygous deletion of *DGCR8* and the schizophrenia network. The results suggest that the levels of influence of these two miRNA mechanisms were at similar levels. Collectively, these findings provide new insights into the pathway from 22q11.2 deletion to expression of schizophrenia. The overlap between the 22q11.2DS miRNA mechanism-related targets and the schizophrenia network suggests that hemizygosity of the 22q11.2 region may have downstream effects that are relevant to the general schizophrenia population. These data also provide further support for the optimistic notion that robust genetic findings in schizophrenia will soon be found to converge on a reasonable number of final pathways (42). For example, Figure 3 and Table S1 in Supplementary Material document genes related to 22q11.2 region miRNA mechanisms that include several involved in glutamatergic and/or dopaminergic pathways, such as *GRIA1* (40). Other implicated genes are key regulators of neurodevelopment, e.g., *FGF2* (72). Genes such as *NRXN1* (73) and *DISC1* (74) have now been shown to be involved in the aetiopathogenesis of schizophrenia through diverse lines of evidence.

### Advantages and limitations

To our knowledge, this is the first study of the potential consequences of reduced gene dosage effects of the collective miRNA-related content in the 22q11.2 deletion region. Animal model and human miRNA studies performed to date relating to the 22q11.2 deletion had primarily focused on *DGCR8* and, to a lesser extent, miR-185 (21–24). There is little written about the cluster of the seven validated and putative miRNAs in the 22q11.2 deletion region beyond miR-185. For example, a recent publication listed just four miRNAs in this region (75). Also, miRNAs, including miR-185 and the other six from this region, are not represented on standard RNA expression arrays, and thus are not available for reporting in existing expression databases and papers. These technology-based limitations would be in addition to important issues related to expression differences between specific tissues, brain regions, and developmental stages. There are also species differences, and potentially activity-regulated differences, in expression. For example, in rat hippocampal neuron cultures, miR-185 has recently been shown to be associated with altered expression after NMDA receptor-dependent plasticity changes (76). A further limitation of the current study is the reliance, related to all bioinformatic approaches, on published data. As new expression and other data become available, there will be further opportunities to study the potential contribution of regional miRNA mechanisms to expression of schizophrenia in 22q11.2DS. We note that *DGCR8* has activities that are miRNA independent (77, 78), that could be additive to its roles in miRNA-processing genome-wide.

One limitation of all contemporary miRNA studies is the forced reliance on target gene prediction tools, given that there are limited validated gene target data available, and even then the miRNA expression studies that are the gold standard may be imperfect (79, 80). For that reason, in this preliminary study we did not restrict to validated targets. We also did not add validated targets. As an example, *HTR2C*, one of seven genes dramatically decreased in *Dgcr8*^±^ mutant mice hippocampus and previously shown to be decreased in prefrontal cortex in schizophrenia (24), though present in our network, was not a predicted *DGCR8* target using our methods. Also, miRNAs are being identified daily, and the prediction tools are limited by the data available on these miRNAs and their gene targets. Different gene target prediction software can produce differing sets of predicted target genes. For example, Forstner and colleagues (81) used the Molecular Signatures Database 3.1 to derive 124 targets of miR-185. We elected to employ a strategy based on two distinct prediction tools for miRNA target prediction, and generation of averaged percentile-transformed scores. Although imperfect, the integration of more than one prediction method tends to balance out the precision and recall, arguably resulting in better accuracy and coverage of predictions (19, 82).

In the absence of any single widely accepted schizophrenia gene list or network, we generated a network using data derived from a published CNV and GWAS study (40, 41) together with genes relevant to brain function. This network was designed to be free of bias from 22q11.2 deletion effects. The relatively few candidate genes for schizophrenia highlighted in the recent large-scale PGC GWAS was in part a consequence of the high threshold for genome-wide significance (40). The specific approach that led to the selection of the 35 genes in that study was not described in detail and thus could not be applied to the other gene list used in this study. In contrast, the list of genes derived from the study of rare CNV (40, 41) was larger, particularly in proportion to the respective sample sizes of the two studies. In this case, many more genes were scrutinized and thus ultimately considered to be of potential relevance to schizophrenia, because of the implicit assumption that any very rare CNV could potentially be a risk factor of moderate or greater effect. Many of these 107 genes had been implicated in other studies of rare copy number or sequence variation (42–54). We applied homogeneous criteria to filter both candidate gene lists, using up-to-date and comprehensive annotations, resulting in a more balanced final ratio of GWAS to rare CNV candidates of about 1:2. Furthermore, the dependence of the network on the initial candidate list was reduced by the inclusion of genes relevant to brain function that interact not only with the schizophrenia candidates but also with other brain-specific genes.

### Future directions

Functional studies could determine if there is an appreciable difference in gene expression in the 22q11.2 region miRNA gene targets highlighted in this study, between those individuals with 22q11.2DS who did and did not develop schizophrenia. As suggested by our functional enrichment mapping results, miRNAs in this deletion region may also play a role in other developmental aspects of the 22q11.2DS phenotype, including congenital heart disease. More generally, our experimental approach could now be applied to miRNAs and their target genes within the genomic extent of diverse large rare CNVs with variable neuropsychiatric and other expression, e.g., duplications and deletions at 16p13.11 (19, 50). Conversely, one could investigate whether other areas of the genome where miRNA density is high are more likely to have rare CNV deemed pathogenic. Interaction partners of schizophrenia candidates included in our network may be helpful to delineate the broader pathways that may be underpinning schizophrenia pathogenesis, and thus could be implicated by future studies (following the principle of “guilt-by-association”) (68). Adding to a focus on variants in the exome, there is recent evidence that rare, non-coding variants have an important impact on expression and complex disease burden (83). While rare variants may have higher prior likelihood of functional impact (83), a miRNA mechanism can embrace both rare and common variants in pathogenesis (27). Using advanced technologies in integrated studies of the transcriptome and genome (83) may contribute to discovering the “hidden heritability” of schizophrenia.

## Conflict of Interest Statement

Linda M. Brzustowicz serves as a consultant for the Janssen Pharmaceutical Companies of Johnson & Johnson, and serves on the Scientific Advisory Board of Motif BioSciences. The other authors have no potential conflicts of interest to declare.

## Supplementary Material

The Supplementary Material for this article can be found online at http://www.frontiersin.org/Journal/10.3389/fneur.2014.00238/abstract

## Footnotes

^1^http://www.bioconductor.org/packages/release/data/annotation/html/org.Hs.eg.db.html

^2^http://www.biocarta.com

^3^http://www.omim.org/

^4^http://www.informatics.jax.org/

^5^http://www.mirbase.org
